# The *Caenorhabditis elegans* interneuron ALA is (also) a high-threshold mechanosensor

**DOI:** 10.1186/1471-2202-14-156

**Published:** 2013-12-17

**Authors:** Jarred Sanders, Stanislav Nagy, Graham Fetterman, Charles Wright, Millet Treinin, David Biron

**Affiliations:** 1 Committee on Genetics, Genomics, and Systems Biology, The University of Chicago, Chicago, IL 60637, USA; 2 The Institute for Biophysical Dynamics, The University of Chicago, Chicago, IL 60637, USA; 3 Department of Physics and the James Franck Institute, The University of Chicago, Chicago, IL 60637, USA; 4 Department of Medical Neurobiology, Institute for Medical Research – Israel-Canada, Hebrew University – Hadassah Medical School, Jerusalem 91120, Israel

**Keywords:** Mechanosensation, Physiology, Neuroethology

## Abstract

**Background:**

To survive dynamic environments, it is essential for all animals to appropriately modulate their behavior in response to various stimulus intensities. For instance, the nematode *Caenorhabditis elegans* suppresses the rate of egg-laying in response to intense mechanical stimuli, in a manner dependent on the mechanosensory neurons FLP and PVD. We have found that the unilaterally placed single interneuron ALA acted as a high-threshold mechanosensor, and that it was required for this protective behavioral response.

**Results:**

ALA was required for the inhibition of egg-laying in response to a strong (picking-like) mechanical stimulus, characteristic of routine handling of the animals. Moreover, ALA did not respond physiologically to less intense touch stimuli, but exhibited distinct physiological responses to anterior and posterior picking-like touch, suggesting that it could distinguish between spatially separated stimuli. These responses required neither neurotransmitter nor neuropeptide release from potential upstream neurons. In contrast, the long, bilaterally symmetric processes of ALA itself were required for producing its physiological responses; when they were severed, responses to stimuli administered between the cut and the cell body were unaffected, while responses to stimuli administered posterior to the cut were abolished.

**Conclusion:**

*C. elegans* neurons are typically classified into three major groups: sensory neurons with specialized sensory dendrites, interneurons, and motoneurons with neuromuscular junctions. Our findings suggest that ALA can autonomously sense intense touch and is thus a dual-function neuron, i.e., an interneuron as well as a novel high-threshold mechanosensor.

## Background

To survive in dynamic or harsh environments, all animals must appropriately modulate their responses to various stimulus intensities. For instance, noxious stimuli are detected by nociceptors, an important class of high-threshold sensory neurons. These, in turn, lead to downstream immediate avoidance responses and enduring self-protective responses that are distinct from responses to milder stimuli [1-5]. Neurons similar to mammalian polymodal nociceptors in both function and molecular determinants have been found across the animal kingdom [6-8]. In the nematode *Caenorhabditis elegans*, the neuron types PVD and FLP have been shown to share many similarities with mammalian and *Drosophila* nociceptors [3,9-13], including a conservation of molecular mechanisms underlying the responses to noxious stimuli [5,6,8,14-19].

**Figure 1:**
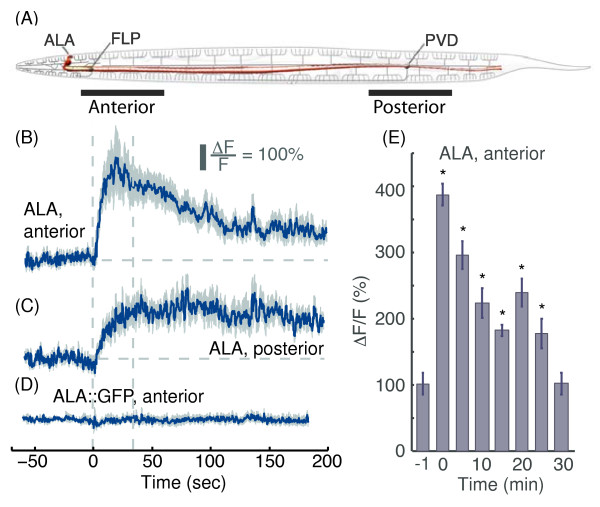
**ALA responds to anterior and posterior picking-touch stimuli. (A)** a schematic drawing of ALA (red), and the proprioceptor neurons PVD and FLP (grey). The locations where anterior and posterior mechanical stimuli were applied are denoted with black bars (schematic adapted from wormatlas.org). GCaMP3 fluorescence levels in ALA before and after administering anterior **(B)** or posterior **(C)** picking-touch stimuli at t=0 with a platinum wire pick. The scale bar represents a 100% deviation from the mean baseline fluorescence. Mean ± s.e.m, N=9-12 animals. **(D)** GFP fluorescence levels under the same conditions as in **(B-C)**. Mean ± s.e.m, N=10 animals. Dashed lines in panels **(B-D)** are provided as a guide to the eye. **(E)** The mean GCaMP fluorescence levels during 20 sec intervals, recorded 1 minute prior to administering an anterior stimulus, immediately after the stimulus, and every 5 minutes thereafter. Mean ± s.e.m, N=10 animals. Asterisks denote a significant difference from the baseline mean fluorescence (p < 0.05).

*C. elegans* neurons are typically classified into three major groups: sensory neurons with specialized sensory dendrites, interneurons, and motoneurons with neuromuscular junctions. However, these groups are not strictly mutually exclusive. For instance, the DVA interneuron was found to be a stretch-sensitive sensory neuron [20]. ALA is a unilaterally-placed single interneuron (Figure 1A). It has a pair of bilaterally-symmetric processes that branch from the soma, and proceed along the left and right sides of the body to the tail region, adjacent to the excretory canals. A third, short process is sent from the soma to the dorsal cord [21]. ALA has been shown to be involved in reducing the velocity of animals, as well as their rate of pharyngeal pumping in an epidermal growth factor-dependent manner [22]. It has also been reported to decrease locomotion in a manner subject to regulation by the CEPsh sheath cells [23].

Here we show that ALA acted as a high-threshold mechanosensor, and that it played a role in a previously described response to intense mechanical stimuli [8]. ALA exhibited physiological responses to both anterior and posterior stimuli, and it was required for the inhibition of egg-laying in response to picking-touch (see Methods). The physiological responses of ALA to anterior and posterior touch were distinct, suggesting that it could distinguish between spatially separated stimuli. In addition, these responses did not require neurotransmitter or neuropeptide release from upstream neurons. However, the bilaterally symmetric processes of ALA itself were required for generating its physiological responses. These results suggest that ALA can autonomously sense picking-touch, but not lower intensity touch stimuli, and is thus a high-threshold mechanosensor.

## Results

### The ALA neuron responded to both anterior and posterior picking-touch stimuli

**Figure 2:**
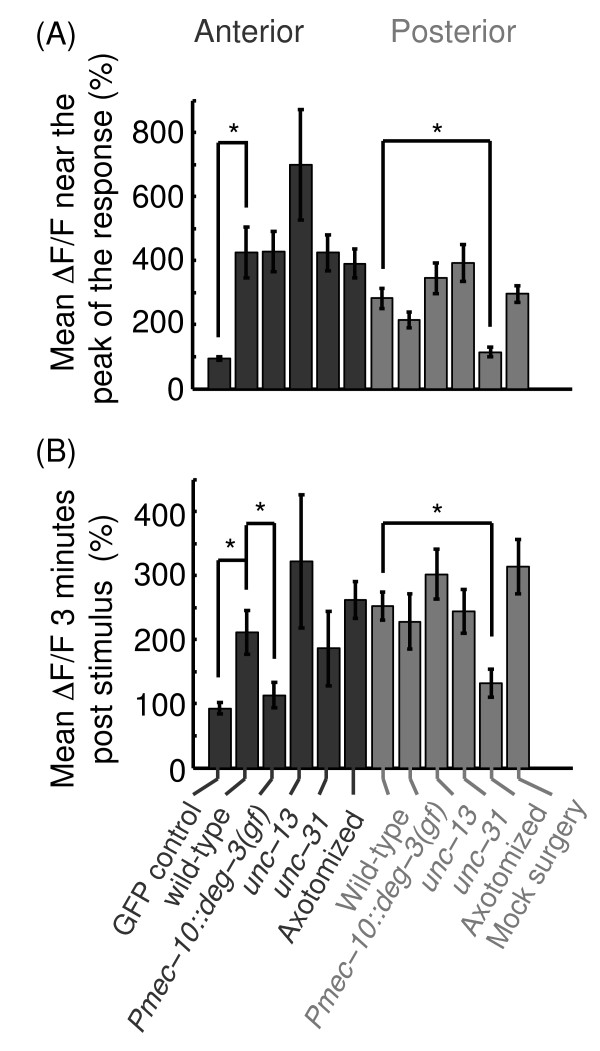
**A comparison of ALA physiological responses to picking-touch. (A)** The mean GCaMP fluorescence during the 20 seconds surrounding peak activity, t=5-25 sec and t = 40–60 sec post-stimulus for anterior and posterior stimuli, respectively. Asterisks denote that the measurements of GFP (control) fluorescence and the post-surgery responses to posterior stimuli were significantly different from the corresponding wild-type responses (p<0.05). **(B)** The mean GCaMP fluorescence at t = 160–180 sec. Asterisks denote that the measurements of GFP (control) fluorescence, the responses in *Pmec-10::deg-3(u662)* animals , and the post-surgery responses to posterior stimuli were both significantly different from the corresponding wild-type responses (p<0.05). All mean fluorescence levels were calculated from the datasets shown in Figures 1, 5, 7, and 8.

After serendipitously observing physiological responses to picking-touch (see Methods) in ALA neurons we sought to characterize these responses. In order to assay the physiological responses of ALA, we expressed the genetically encoded calcium indicator GCaMP3 [24] under the control of the ALA-specific *ver-3* promoter [22,25-34]. Touch stimuli were applied to either the anterior or the posterior region of the animal, and the resulting fluorescence intensity of the cell soma was recorded. We did not observe a response either to gentle-touch or to harsh-touch in ALA neurons (see Methods). Therefore, ALA was not found to be a low-threshold mechanosensor. In contrast, both anterior and posterior picking-touch evoked calcium transients in ALA (Figure 1). In the case of anterior stimuli, GCaMP fluorescence exhibited a 10 sec increase to 400% of the baseline followed by a decline to 200% of the baseline after 100 sec (Figure 1B). Posterior stimuli resulted in a gradual 50 sec increase in fluorescence to 300% of the baseline (Figure 1C). In both cases, GCaMP3 fluorescence did not return to baseline within the 5 minutes of the measurement but sustained similar levels of 200% of the baseline (see also Figure 2). To test how long ALA remained active while minimizing photobleaching, we recorded it for 20 sec every 5 minutes. We found that 30 minutes after an anterior stimulus had been given, GCaMP3 fluorescence was not significantly different than its pre-stimulus baseline (Figure 1E).

**Figure 3:**
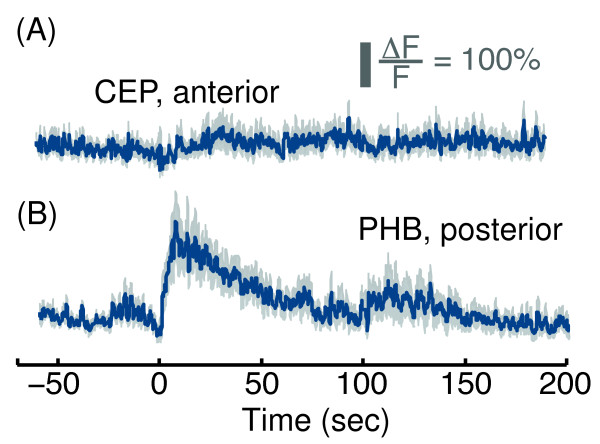
**CEP neurons did not exhibit a detectable response to anterior picking-touch, and PHB neurons responded to posterior picking-touch.** GCaMP3 fluorescence levels in CEPDL/R neurons before and after administering anterior stimulus **(A)** and in PHB neurons before and after administering a posterior stimulus **(B)** at t=0 with a platinum wire pick. The scale bar represents a 100% deviation from the mean baseline fluorescence. Mean ± s.e.m, N=10 animals.

To control for motion artifacts, the fluorescence of *Pver-3*::GFP was assayed for anterior picking-touch under identical conditions and did not deviate from its baseline level (Figure 1D). A negative control (Figure 3A) was provided by the absence of detectable physiological responses in the dopaminergic CEPD/L head neurons [35] despite the fact that the ALM neurons were previously shown to indirectly activate CEP in response to an appropriate anterior body touch [36]. As a positive control, we expressed GCaMP3 in the phasmid chemosensory PHB neurons. Although the physiological responses of PHB neurons to harsh touch have not been previously reported, they were implicated in posterior harsh touch in a laser ablation study [32]. In response to posterior picking-touch, we observed a 3-fold increase in GCaMP fluorescence in PHB, which returned to baseline level after 100 sec (Figure 3B). These results suggest that ALA either received input from an upstream high-threshold mechanosensor or sensed picking-touch autonomously. In addition, the differences in the temporal dynamics of the responses to anterior and posterior picking-touch suggested that the neuron sensing the stimulus was able to discriminate between different spatial positions of stimuli along the body of the animal.

### A mutation that impairs ALA differentiation abolished the reduced egg-laying response to picking-touch

The commonly performed act of transferring a single animal to a new plate using a platinum wire pick inadvertently delivers an intense mechanical stimulus to the animal and evokes a characteristic behavioral response. It was previously shown that this mechanical stimulus leads to PVD- and FLP-mediated egg-laying inhibition lasting 30 minutes [8]. The same procedure was also shown to affect locomotion on a timescale of several minutes [37]. In addition, posterior gentle touch was shown to lead to increased intervals between calcium transients of the HSN egg-laying neurons [38]. We thus asked whether ALA might have a role in mediating these behavioral responses. To address this question, we compared the suppression of egg-laying in wild-type animals to animals mutant for *ceh-17*, a gene encoding a paired-like homeodomain transcription factor.

**Figure 4:**
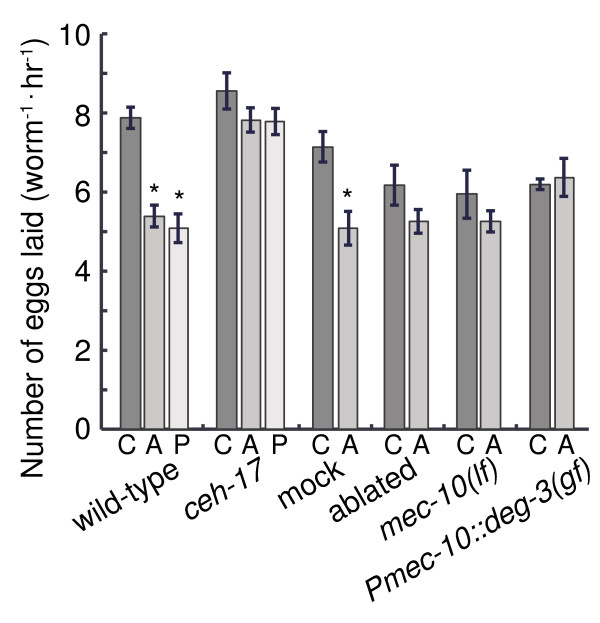
**ALA is required for the suppression of the egg-laying response to picking-touch.** The number of eggs laid per animal, per hour, in animals exposed to anterior or posterior picking-touch stimuli (“A” and “P” respectively) and in control animals (“C”). Mean ± s.e.m, N=4-13 assay plates (see Methods). Asterisks denote statistically significant comparisons (p<0.05).

CEH-17 is expressed in ALA and the cholinergic SIA head neurons and is involved in longitudinal axonal navigation and ALA differentiation [22,39]. Specifically, CEH-17 was shown to regulate the expression of the tyrosine phosphatase-like receptor gene *ida-1*, which is required for ALA neuropeptide release, and *ceh-17* mutants exhibited shortened ALA processes [38,40]. Since the ALA neuron has two bilaterally symmetrical processes that extend from the cell body to the tail of the animal [21], we assayed responses to anterior and posterior stimuli separately. In agreement with previous observation, in our hands both anterior and posterior picking-touch led to a 35% reduction in the number of eggs that were laid by wild-type animals during a 3-hour period. In contrast, in *ceh-17(np1)* mutants the inhibition of egg-laying in response to both anterior and posterior stimuli was completely abolished (Figure 4). In agreement with previous findings [39], we did not observe differences between the locomotive responses of wild-type animals and *ceh-17* mutants to picking-touch (data not shown).

### Laser ablation of ALA abolished the reduced egg-laying response to picking-touch

To specifically test whether ALA was required for the egg-laying inhibition response to picking-touch, we assayed animals in which the ALA neuron was laser ablated during the L4 larval stage. As opposed to the mock-ablated control animals, ALA-ablated animals did not exhibit suppression of the egg-laying response (Figure 4). However, the egg-laying rate of unperturbed post-surgery animals was variable, with a lower mean (although not significantly: p = 0.114 by one-way ANOVA) than the corresponding rate in mock-ablated controls. This variability may be indicative of unspecific damage of the surgery that could not be avoided. We obtained similar results (Figure 4) for two strains in which behavioral responses to harsh touch were abolished [6,8]: mutants for the *mec-10* gene, encoding an amiloride-sensitive sodium channel protein of the DEG/ENaC family that is required for *C. elegans* touch sensation [3,9,11-13], and animals where a degeneration-causing, constitutively active nicotinic acetylcholine receptor (nAChR) channel subunit, *deg-3(u662)*, was expressed under a *Pmec-10* promoter [8,14,16], driving expression in and degeneration of PVD, FLP and the six touch receptor neurons [1,11]. The suppression of egg-laying was not significant in *mec-10(tm1552)* mutants (p = 0.193 by one-way ANOVA) or *Pmec-10::deg-3(u662)* animals (p = 0.732 by one-way ANOVA) [2,3,6,32,41]. Taken together with the responses of *ceh-17* mutants, our results suggest that ALA is required for the inhibition of egg-laying in response to picking-touch, but not for immediate avoidance responses.

### The harsh-touch sensory neurons were not required for the physiological response of ALA to picking-touch

**Figure 5:**
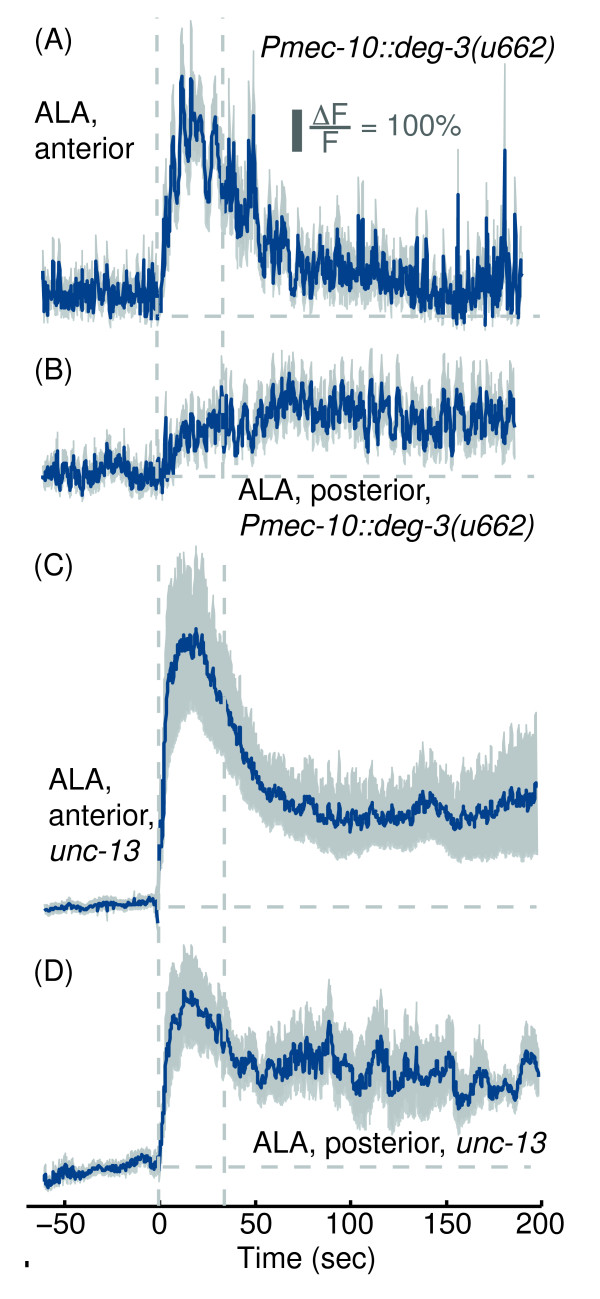
**PVD, FLP and the harsh touch response neurons, as well as neurotransmitter release, were not required for the responses of ALA to picking-touch.** GCaMP3 fluorescence levels in ALA neurons of animals lacking the PVD, FLP and six harsh touch neurons **(A-B)** and in *unc-13(e51)* mutants **(C-D)**. The scale bar represents a 100% deviation from the mean baseline fluorescence. Mean ± s.e.m, N=10-11 animals. Dashed lines are provided as a guide to the eye.

The interneuron ALA has not been previously reported to act as a mechanosensory neuron, but the effects on egg-laying of ablating it were similar to those of ablating known mechanosensors. Moreover, although synapses between ALA and PVD have not been previously found, the elongated processes of ALA and the primary dendrites of PVD are in close proximity [3,4,21]. It was thus possible that ALA transduced signals from known mechanosensory neurons, so we asked whether the physiological responses that we observed were dependent on input from them. To test this, we crossed our P*ver-3*::GCaMP3 reporter into transgenic animals expressing the degeneration-causing nAChR channel subunit, *deg-3(u662)*, in PVD, FLP and the six harsh touch neurons [7,14,16]. In agreement with previous results [8,10,11], these animals failed to respond to standard harsh touch stimuli (data not shown). In addition, the physiological responses of their ALA neurons to posterior picking-touch were unaffected by the genetic ablation (Figures 5B and 2). However, in response to anterior picking-touch, GCaMP fluorescence in ALA returned to its baseline level after 2 minutes in these transgenics (Figure 5A and 2), in contrast to the wild-type prolonged response to the same stimulus. We concluded that the responses of ALA did not require the function of PVD, FLP and the six touch receptor neurons, although a sub-group of these neurons may have a role in sustaining the responses to anterior stimuli.

### The physiological response of ALA to picking-touch did not require neurotransmitter release

Although physiological responses in ALA did not require known mechanosensory neurons, it was still possible that the responses were dependent on input from other pre-synaptic partners of ALA. To answer this question we crossed our P*ver-3*:GCaMP3 reporter into an *unc-13(e51)* mutant background, where synaptic vesicle exocytosis is essentially eliminated [6,8,15,17-19,42,43]. Anterior and posterior picking-touch evoked rapid and long-lasting calcium transients in the ALA neurons of *unc-13(e51)* mutants (Figures 5C-D and 2). The peaks of *unc-13(e51)* responses to anterior stimuli were highly variable, with a coefficient of variance of 0.78 (Figure 2A), but were not significantly different from wild-type (p = 0.103 by one-way ANOVA). In contrast to wild-type, the rise time of the responses to posterior stimuli was rapid (5 sec) in the mutant animals, and their dynamics were similar to those of responses to anterior stimuli. These results suggested that synaptic input from neurotransmitter release was not necessary for producing the responses of ALA to picking-touch. However, such input may contribute to more subtle aspects of regulating these responses (similar to the case of the *Pmec-10::deg-3(u662)* transgenic background) such as the initial dampening of responses to posterior stimuli.

### Presynaptic partners of ALA responded to anterior picking-touch

**Figure 6:**
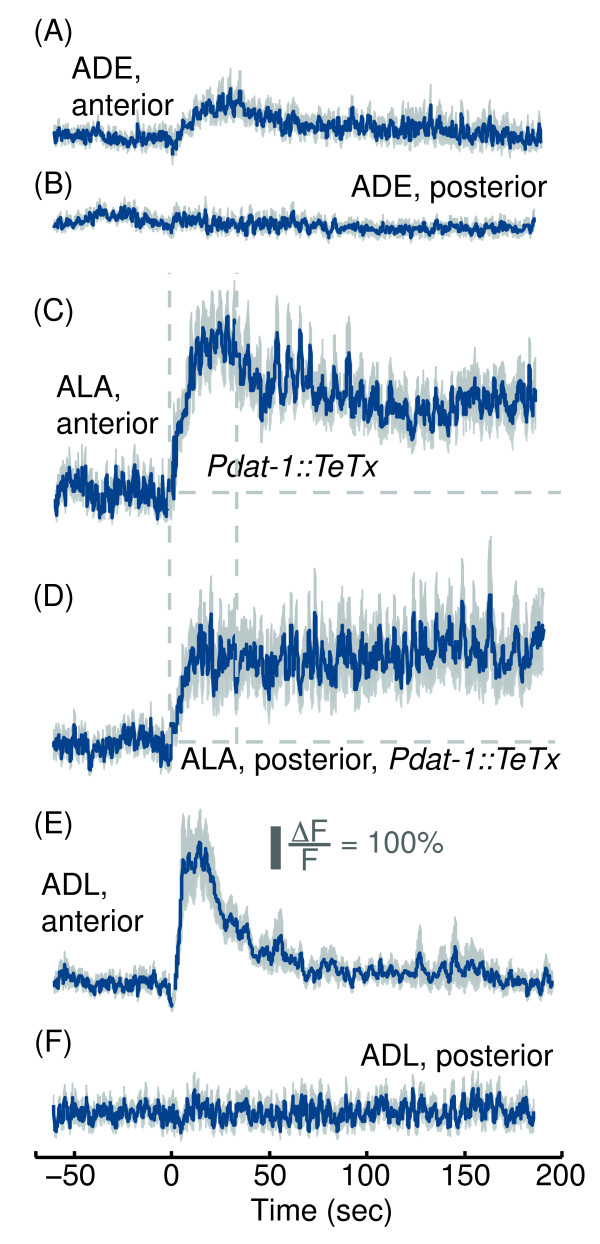
**The sensory neurons ADE and ADL, both presynaptic partners of ALA, responded to anterior but not to posterior picking-touch.** GCaMP3 fluorescence levels in ADE neurons **(A-B)**, ALA neurons in animals expressing the tetanus toxin light chain in their dopaminergic neurons **(C-D)** and in ADL neurons **(E-F)**. In all cases responses to both anterior and posterior stimuli are shown. Mean ± s.e.m, N=10 animals. Dashed lines in panels **(C-D)** are provided as a guide to the eye.

Since blocking neurotransmitter release modulated the kinetics of the responses of ALA, we asked whether its presynaptic partners responded to picking-touch. Specifically, we focused on two presynaptic sensory neurons: ADE, previously implicated in anterior touch responses by laser ablation experiments [32,36,44-46], and ADL, a polymodal nociceptive neuron [47-50]. We expressed GCaMP3 in ADE (and all of the dopaminergic neurons) and ADL using the *Pdat-1*[21,51] and *Psrh-220*[22,52] promoters, respectively. ADE neurons exhibited a slowly rising response to anterior but not posterior picking-touch, which peaked after 40 sec and returned to baseline after 5 min (Figure 6A-B). However, abolishing vesicle exocytosis in all dopaminergic neurons by driving expression of the tetanus toxin light-chain with the *dat-1* promoter [23,53,54] did not significantly alter the responses of ALA to picking-touch (Figure 6C-D). Consistent with previous reports [8,50], ADL neurons responded to the onset of blue light. However, this response decayed after 4 minutes of continuous illumination such that the animal could be assayed approximately 10 minutes after initially being exposed to light. ADL neurons also responded to anterior, but not posterior picking-touch, and their response decayed to baseline after 70 sec (Figure 6E-F). Taken together with the responses observed in *unc-13* mutants, these results indicated that synaptic input from ADE and ADL did not contribute to the responses of ALA to picking-touch.

ADL, a presynaptic partner of ALA, has been shown to be a chemosensory neuron that plays a role in avoidance behavior in the presence of volatile repellents such as octanol [48,55]. Ablating ADL has been shown to increase the latency of responses to octanol off food, but not on food [8,56]. We thus sought to test whether ALA might be mediating octanol avoidance in addition to responses to picking-touch. To answer this question, we dipped an eyebrow hair in 30% octanol and presented it in front of the nose of P*ver-3*::GCaMP3 animals. We observed reversals characteristic of the avoidance response of *C. elegans* in both wild-type animals and *ceh-17* mutants, with latencies of 4 sec in both genetic backgrounds, both on and off food (data not shown). Consistent with the behavioral assay, the repellent did not evoke detectable calcium transients in the ALA neuron (data not shown). Taken together, these results suggested that ALA did not mediate octanol avoidance.

### The physiological responses of ALA to picking-touch were independent of neuropeptide release

**Figure 7:**
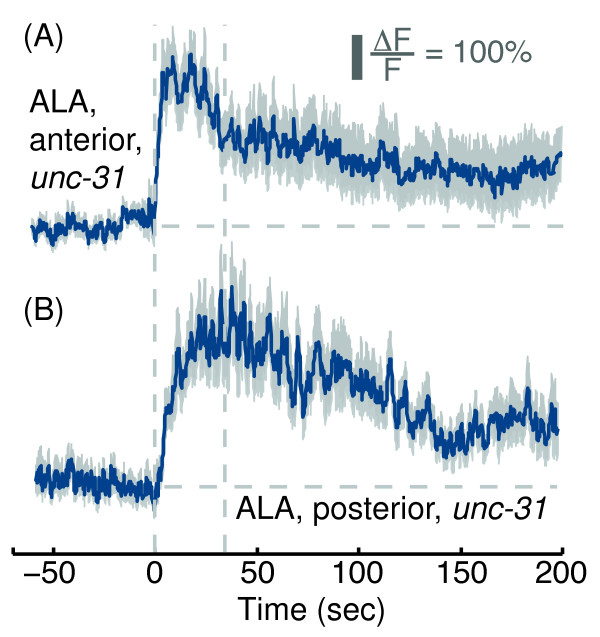
**Neuropeptide release was not required for the responses of ALA to picking-touch.** GCaMP3 fluorescence levels in ALA neurons of *unc-31(e169)* mutants responding to anterior **(A)** and posterior **(B)** picking-touch. Mean ± s.e.m, N=10 animals. Dashed lines are provided as a guide to the eye.

Although input from neurotransmitter release of pre-synaptic partners of ALA was not required for producing its physiological responses to picking-touch, it could be the case that peptidergic signaling from an unknown mechanosensory neuron was required. The *unc-31* gene encodes an ortholog of the mammalian CAPS protein and is required for dense-core vesicle but not synaptic vesicle exocytosis of neuropeptides [32,57-59]. Thus, we crossed our P*ver-3*::GCaMP3 reporter into an *unc-31(e169)* mutant background, where dense-core vesicle exocytosis is practically eliminated. Anterior picking-touch evoked wild-type-like responses in the ALA neurons of *unc-31(e169)* mutants, while posterior touch evoked responses that appeared slightly enhanced during the first 50 sec post-stimulus, but not significantly so (Figures 7 and 2: p = 0.998 and p = 0.162 by one-way ANOVA for anterior and posterior stimuli, respectively). These results suggested that neuropeptide release was not required for evoking or sustaining the responses of ALA to picking-touch.

### The elongated processes of the ALA neuron are required for its physiological response to picking-touch

**Figure 8:**
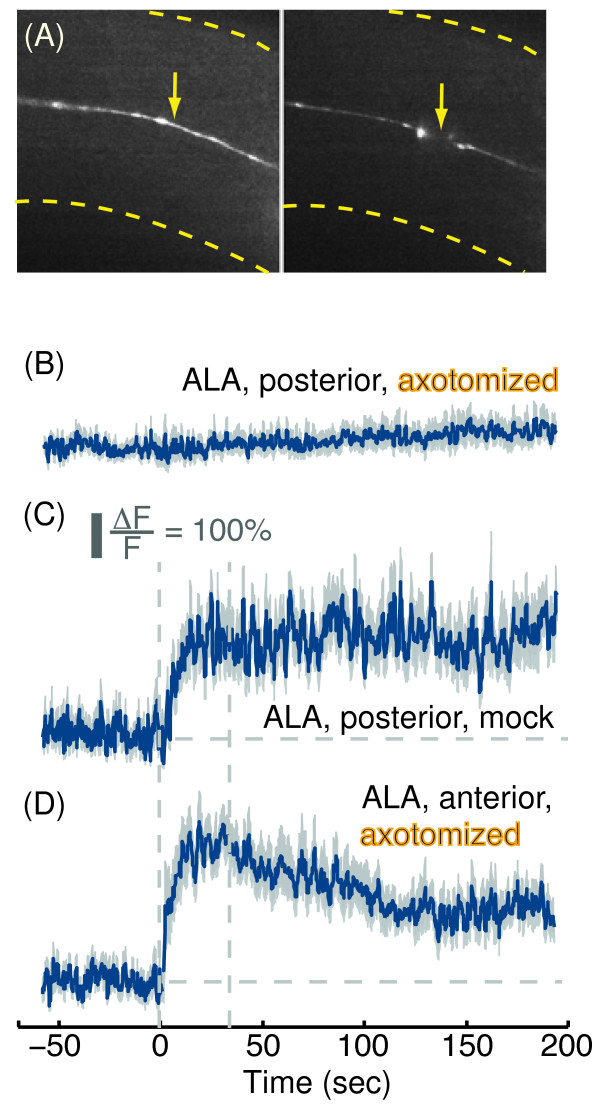
**Severing both of the elongated processes of ALA abolished its responses to picking-touch stimuli that were positioned posterior to the cut. (A)** A representative image of a single ALA process expressing GCaMP3 before (left) and after (right) it was severed. **(B-C)** GCaMP3 fluorescence levels in ALA neurons before and after a picking-touch stimulus was administered posterior to the cut in operated animals **(B)** and in mock-operated controls **(C)**. Mean ± s.e.m, N_operated_=8 and N_mock_=10 animals. **(D)** Same as **(B)**, but for stimuli that were administered anterior to the cut, i.e., between the cut and the ALA cell body. Mean ± s.e.m, N_operated_=11 animals. Dashed lines in panels **(C-D)** are provided as a guide to the eye.

We reasoned that if ALA was autonomously sensing picking-touch, then it would likely require its long, bilaterally symmetric processes to do so. If this is the case, severing the elongated processes between the nerve ring and the vulva should abolish ALA responses, at least to stimuli that are posterior to the cut. We used a femtosecond pulsed laser to sever ALA processes [32,57,60-63] in wild-type L4 larvae expressing our reporter, *Pver-3::GCaMP3*, and assayed them as young adults 24 hours post-surgery (Figure 8A). We found that the physiological responses of ALA to posterior picking-touch downstream from the cut were completely abolished in axotomized animals and unaffected in the mock-axotomy controls (Figures 8B-C and 2). In contrast, ALA responses to anterior picking-touch, upstream from the cut, were unaffected by the surgery (Figures 8D and 2). The wild-type like responses to anterior stimuli suggested that the part of the cell upstream from the cut, including the soma, was not damaged by the surgery – an internal control that is typically difficult to obtain. These results supported the conclusion that ALA autonomously sensed picking-touch stimuli and that its long processes were required for this function. In addition, they suggested that ALA could discriminate between different spatial positions of stimuli along the body.

## Discussion and conclusions

In this study we show that the ALA interneuron of the nematode *C. elegans* exhibits physiological responses to picking-touch and that it is required for a stereotypical response to this stimulus, a suppression of egg-laying [8,32,38]. The physiological responses of ALA did not require input from presynaptic release of neurotransmitters, nor did they require neuropeptide release. When the elongated, bilaterally symmetric processes of ALA were axotomized, the physiological responses to stimuli that were on the anterior side (upstream) of the cut persisted, while the responses to stimuli on the posterior side (downstream) of the cut were eliminated. ALA is known to form several electrical synapses, all of which are located in the anterior sections of its processes, i.e., in vicinity of the cell soma [21,64]. As a result, our axotomy experiments rule out the possibility that input from the known gap junctions of ALA was capable of producing the observed responses. Taken together, our findings suggest that ALA can sense picking-touch stimuli autonomously, i.e., that it is a high-threshold mechanosensor.

The ALA neuron could discriminate between spatially separated stimuli along the body of the animal. Three observations are consistent with a model in which (as of yet unknown) molecular sensors are distributed along the processes of ALA: (1) the temporal dynamics of the responses to anterior and posterior stimuli were distinct, (2) neurotransmitter and neuropeptide release affected anterior and posterior responses differently, and, most importantly, (3) when the elongated processes of ALA were severed the responses to stimuli applied posterior but not anterior to the cut were abolished. Measuring the receptive field map of ALA using more accurately localized stimuli could provide insight into the spatial differentiation of this mechanosensor.

The physiological responses that we observed in ALA were unusually prolonged as compared to typical responses of *C. elegans* sensory neurons [36,61,62,65-70]. Application of menthol, a noxious stimulus, can produce similarly prolonged calcium transients in mammalian nociceptors [71]. However, axonal injury in mammalian and invertebrate preparation can also produce long-lasting calcium responses [72,73]. Could the physiological and behavioral responses that we observed result from a compression or strain injury inflicted on ALA by picking-touch? Several considerations suggest that this is unlikely: (1) picking was used to transfer “control” animals to assay plates, as well as for their maintenance, such that a potential injury in ALA would be required to heal on the timescale of an hour in order to allow for the higher egg-laying rate in the absence of the recurring stimulus, (2) ALA is not at all unique in being located peripherally or having elongated processes; similar anatomical features are characteristic of many touch receptor, proprioceptor and motor neurons in *C. elegans*[21], (3) egg-laying was not suppressed in response to anterior picking-touch in *ceh-17* mutants, where the anterior half of the ALA process was typically present; moreover, the developmental defect in ALA neurons in *ceh-17* mutants did not suppress egg-laying, and (4) in *C. elegans*, axotomy induced calcium dynamics in the soma of the touch receptor neuron ALM exhibited a strong dependence on the lesion distance, effectively vanishing when the axon was injured merely 40 μm from the soma [73]. Thus, explaining the reported responses as a result of injury would require multiple unsupported assumptions regarding the unique nature of ALA, such as an unexplained enhanced vulnaribility to compression, an ability to heal rapidly, an effect on egg-laying in response to injury that is distinct from a non-specific loss of function, and post-injury calcium dynamics dissimilar from those of a *C. elegans* touch receptor neuron.

Our findings associate the responses of ALA to picking-touch with the enduring inhibition of egg-laying, but not with an immediate avoidance response. Since enduring behavioral responses have been shown to depend on neuropeptides in previous studies [8,35], we hypothesize that this could also be the case here. Based on its anatomical features, it has been suggested that ALA may be a neurosecretory neuron [21,36,74]. It has been shown that ALA is required for the regulation by epidermal growth factor (EGF) signaling of feeding and locomotion patterns, and that feeding defects caused by overexpression of the EGF-like peptide LIN-3 are suppressed by a mutation in the gene encoding UNC-31/CAPS [22,32]. In addition, the tyrosine phosphatase-like receptor gene *ida-1* was shown to be expressed in ALA [8,40,75]. IDA-1 was demonstrated to be important for dense-core vesicle cargo release; it acts genetically in the signaling pathway of *unc-31* (encoding the UNC-31/CAPS protein) suggesting that it has a role in the trafficking of dense core vesicles and/or in their cargo release [37,76]. Importantly, CEH-17 was shown to regulate the expression of *ida-1* in ALA [38,40], such that neuropeptide release from ALA would be expected to be impaired in *ceh-17* mutants. Taken together, these findings and the results presented here are consistent with the idea that ALA may use peptidergic signaling to communicate to its downstream targets.

Several *C. elegans* neurons, such as the proprioceptors DVA, AVG, and PVR, were originally classified as interneurons and later found to also function as sensory neurons. In addition, interneurons functioning also as sensory neurons have been described in other species, e.g. the B51 neuron in *Aplysia californica*[77,78]. However, this study was the first to associate the role of a mechanosensor with the interneuron ALA, thus demonstrating that it is a dual-function neuron. The roles of neuropeptides in modulating the repertoire of enduring responses, as well as the degree to which these roles may be either evolutionarily conserved, remain to be understood.

## Methods

### Strains

Wild-type, transgenic, and mutant *C. elegans* strains were maintained and cultivated with OP50 bacteria according to standard protocols [55]. The following strains were used: wild-type strain N2, INV21001 N2;Ex[P*ver-3*::GCaMP3], INV21002 N2;Ex[P*gpc-1*::GCaMP3], INV21003 N2;Ex[*Psrh-220*::GCaMP3], INV21004 N2;Ex[*Pdat-1*::GCaMP3], INV54001 N2;Ex[*Pdat-1*::TeTx; *Pver-3*::GCaMP3], INV54002 *egl-3(n150)V*; Ex[*Pver-3*::GCaMP3], INV54003 *unc-13(e51)*; Ex[*Pver-3*::GCaMP3], INV54004 *unc-31(e169)*; Ex[*Pver-3*::GCaMP3] and INV54005 N2;Is[*Pmec-10::deg-3(u662)*]; Ex[*Pver-3*::GCaMp3]. The OS5513 N2; Ex[*Pver-3*::GFP] and the IB16 *ceh-17(np1)I* strains were a gift from Menachem Katz of Shai Shaham’s laboratory (Rockefeller University).

### Picking-, harsh- and gentle-touch stimuli

The routine maintenance procedure of picking animals entails applying pressure with a platinum wire covered with a thin sticky layer of bacteria, a procedure performed on the order of 10,000 times a year by a typical experimenter. Although it is difficult to obtain quantitative characterizations of hand-delivered mechanical stimuli, estimates of the pressure applied by a platinum wire pick were previously obtained using an NGM test plates placed on an analytical balance [32]. By this measure, in our hands, picking-touch was estimated to involve pressures that were 10-fold larger than those associated with the standard harsh-touch stimulus. Thus, *C. elegans* researchers commonly apply three distinct tiers of touch stimuli: (1) gentle touch – the weakest intensity tier, e.g., brushing the animal with an eye-lash pick, (2) harsh touch – an intermediate intensity tier, e.g., probing with a platinum wire or a glass rod, and (3) picking touch – the highest intensity tier in routine use [32,57,60]. In addition, picking-touch was previously shown to produce a behavioral response – a suppression of the rate of egg-laying [8]. In our assays, a picking-touch stimulus was delivered in the same manner as in routine picking, but without the thin bacteria layer that would facilitate the lifting of the animal from the substrate. The duration of the stimulus was approximately one second. Thus applied, this common procedure was not previously reported to result in sustained tissue damage, and our data suggests that it did not injure the ALA neuron. To test responses to lower intensity mechanical stimuli, we delivered standard harsh-touch and gentle-touch stimuli using a platinum wire and an eyelash pick, respectively.

### Octanol avoidance assay

Avoidance of 30% 1-octanol was assayed as previously described [48,56]. In brief, an eyelash hair was attached to a Pasteur pipette, dipped in octanol, and presented in front of a forward moving animal without touching it. The amount of time it took the animal to initiate backward movement was determined by a handheld audible timer. Two well-fed animals were placed on fresh NGM plates (either with or without a lawn of bacterial food) 30 minutes prior to each assay. Each animal was tested 7–10 times, with an interval of at least 2 minutes between successive tests.

### Egg-laying assay

Animals were synchronized and assayed at 70 hours post-hatching unless noted otherwise. Animals were placed in triplets on a 3 cm diameter NGM plates at 20°C 20 minutes prior to the beginning of the assay. Picking-touch stimuli were delivered manually to each individual animal every 20 minutes during a 3 hours period. The adults were then removed and the number of eggs per plate was counted. To simplify egg-counting, each assay plate was seeded with a small drop of OP50 bacteria at its center.

### Physiological imaging in freely behaving animals

Animals were manually synchronized by transferring 10 gravid adults to fresh NGM plates and restricting the duration of egg-laying to two hours. The adults were then removed, and the embryos were grown at 20°C until young adulthood. One hour prior to testing, young adult animals expressing the appropriate marker were transferred to a fresh standard NGM plate (6 cm in diameter) spread with a thin layer of OP50 bacteria. Picking-touch stimuli were delivered manually to freely moving animals using a platinum-iridium wire (0.2 mm diameter, 99.9% purity from Alfa Aesar) attached to a glass Pasteur pipette [32]. Anterior and posterior stimuli were applied to the farthest quartiles of the animal body, respectively. A single stimulus was delivered per animal. Calcium imaging of each worm was performed at a magnification of 11.5× for 1 minute prior to the stimulus and 4 minutes post-stimulus. Alternatively, in order to capture the slow decay of the signal, 20 seconds of imaging were performed every 5 minutes for 30 minutes. Images were binned 4×, and captured at 5 Hz using a cooled CCD camera (Photometrics CoolSNAP HQ2, Tucson, AZ), an Olympus SZX16 stereomicroscope equipped with an SDF PLAPO 1XPF objective (Olympus America Inc., Center Valley, PA), and Micro-manager [79]. Animals were tracked manually during the assay and image analysis was performed using custom MATLAB scripts (Mathworks, Inc. Natick, MA).

### Axotomy and ablations

Ablations and axotomy were performed on L4 larvae, immobilized with 10 μM levamisole on a 2% agarose pad, using a Ti:sapphire femtosecond laser, as previously described [61-63]. The transgenes *Pver-3*::GFP and *Pver-3*::GCaMP3 were used to identify ALA neurons for ablations and axotomy, respectively.

## Competing interest

On behalf of the authors of the manuscript, I wish to state that the authors declare no financial or non-financial competing interests (DB).

## Authors’ contribution

JS carried out the molecular, behavioral, and physiological studies and drafted the manuscript. GF carried out behavioral studies. SN and MT participated in the molecular work. CR participated in the data analysis. DB conceived of the study, and participated in its design and coordination and helped to draft the manuscript. All authors contributed to writing and editing of drafts, and approved the final manuscript.
